# Patient-derived xenograft (PDX) tumors increase growth rate with time

**DOI:** 10.18632/oncotarget.6919

**Published:** 2016-01-14

**Authors:** Alexander T. Pearson, Kelsey A. Finkel, Kristy A. Warner, Felipe Nör, David Tice, Manoela D. Martins, Trachette L. Jackson, Jacques E. Nör

**Affiliations:** ^1^ Department of Internal Medicine, University of Michigan School of Medicine, Ann Arbor, MI, USA; ^2^ Department of Restorative Sciences, University of Michigan School of Dentistry, Ann Arbor, MI, USA; ^3^ Department of Oral Pathology, Universidade Federal do Rio Grande do Sul, Porto Alegre, RS, Brazil; ^4^ MedImmune, Gaithersburg, MD, USA; ^5^ Department of Mathematics, University of Michigan School of Literature, Sciences, and the Arts, Ann Arbor, MI, USA; ^6^ Department of Otolaryngology, University of Michigan School of Medicine, Ann Arbor, MI, USA; ^7^ Department of Biomedical Engineering, University of Michigan College of Engineering, Ann Arbor, MI, USA; ^8^ Comprehensive Cancer Center, University of Michigan, Ann Arbor, MI, USA

**Keywords:** mathematical modeling, tumor growth, mouse models, head and neck squamous cell carcinoma, adenoid cystic carcinoma

## Abstract

Patient-derived xenograft (PDX) models are frequently used for translational cancer research, and are assumed to behave consistently as the tumor ages. However, growth rate constancy as a function of time is unclear. Notably, variable PDX growth rates over time might have implications for the interpretation of translational studies. We characterized four PDX models through several *in vivo* passages from primary human head and neck squamous cell carcinoma and salivary gland adenoid cystic carcinoma. We developed a mathematical approach to merge growth data from different passages into a single measure of relative tumor volume normalized to study initiation size. We analyzed log-relative tumor volume increase with linear mixed effect models. Two oral pathologists analyzed the PDX tissues to determine if histopathological feature changes occurred over *in vivo* passages. Tumor growth rate increased over time. This was determined by repeated measures linear regression statistical analysis in four different PDX models. A quadratic statistical model for the temporal effect predicted the log-relative tumor volume significantly better than a linear time effect model. We found a significant correlation between passage number and histopathological features of higher tumor grade. Our mathematical treatment of PDX data allows statistical analysis of tumor growth data over long periods of time, including over multiple passages. Non-linear tumor growth in our regression models revealed the exponential growth rate increased over time. The dynamic tumor growth rates correlated with quantifiable histopathological changes that related to passage number in multiple types of cancer.

## INTRODUCTION

In recent years, the use of patient-derived xenograft (PDX) models generated from surgically implanted tumor fragments into immunodeficient mice has increased substantially, particularly for developmental therapeutic studies. Accurate preclinical models are an essential component to performing translational cancer research, including discerning molecular pathways of oncogenesis and evaluating therapeutics. Tumor cell lines have long existed as a convenient platform for investigation, and numerous cell lines have been well characterized [1]. However, cell line-derived xenograft tumors suffer a lack of predictable relationship between therapeutic responses in preclinical models when compared to responses in human trials [2] and do not accurately recapitulate the tumor microenvironment. Indeed, PDX models were developed in the 1980s to improve the translatability of laboratory results to clinical oncology [3-5]. They typically differ from other xenograft platforms in the fact that cells are never exposed to *in vitro* culture conditions. PDX models have been established for a wide variety of tumor histopathological types, including head and neck cancer [6]. The understanding of potential changes in PDX tumor growth over time is critical for the interpretation of data generated through the use of these models.

Correlations between histopathological and genotypic characteristics of the original patient samples and PDX models have been described in a number of tumor types [7-9]. In addition, the correlation between original human tumor therapeutic response and the response in PDX derived from these same patients has been similarly shown in a number of tumor types [6]. PDX models grown over multiple passages maintain a correlated gene expression profile [10, 11]. In addition, the stability of drug response in PDX models over serial passaging has been described [10]. However, early evidence supports that antineoplastic treatment responses have decreasing consistency at higher passages (unpublished data). One potential reason for these changes is the human to murine transition of tumor-associated stromal tissue in the PDX models [12, 13]. Notably, greater tumor-take rates, and decreased time between passages have been observed [10], but so far these changes have not been quantified or characterized. Further description of predictable passage-related changes within PDX models will allow improved interpretation of results.

Several quantitative methods for analysis of xenograft growth data have been proposed. The Wilcoxon-Mann-Whitney test [14] and analysis of variance (ANOVA) [15] are frequently used to analyze xenograft tumor size differences between groups at a given time point, but these methods ignore data from all other collected time points. Methods applied to incorporate longitudinal data include repeated-measures ANOVA [16], linear mixed model regression [17] and Friedman repeated-measures ANOVA on ranks [18]. A number of Bayesian approaches have also been developed to more accurately describe complex tumor size behaviors under different treatment conditions [19-22]. However, no methods have been developed to evaluate longitudinal xenograft tumor growth information across multiple *in vivo* passages.

Here, we evaluate data generated during the establishment of PDX models for head and neck squamous cell carcinoma (SCC) and salivary gland adenoid cystic carcinoma (ACC). We propose new methods to combine tumor size information over multiple *in vivo* passages. This allows for tumor growth rate interrogation over time periods exceeding the life span of murine hosts. We observed that the growth rate increased over time in both SCC and ACC models in the absence of therapeutic intervention. These growth rates mirrored blinded pathological ratings of histopathological features taken from different tumor passages. The SCC models had increased nuclear pleomorphism, decreased stromal proportion, and reduced inflammatory cell infiltration over passages. We also observed that our ACC models experienced a significant shift in overall histopathological pattern over time. Changes in the number of mitotic figures, nuclear size variability, cytoplasm quantity, nucleoli characteristics, and chromatin quantity were observed as a function of passage. Importantly, the reduced time between passages was a phenomenon shared between both tumor types evaluated here. Understanding these changes is necessary to enable accurate interpretation of data generated from PDX models.

## RESULTS

### PDX tumor models display enhanced growth rate with increased *in vivo* passage

**Figure 1:**
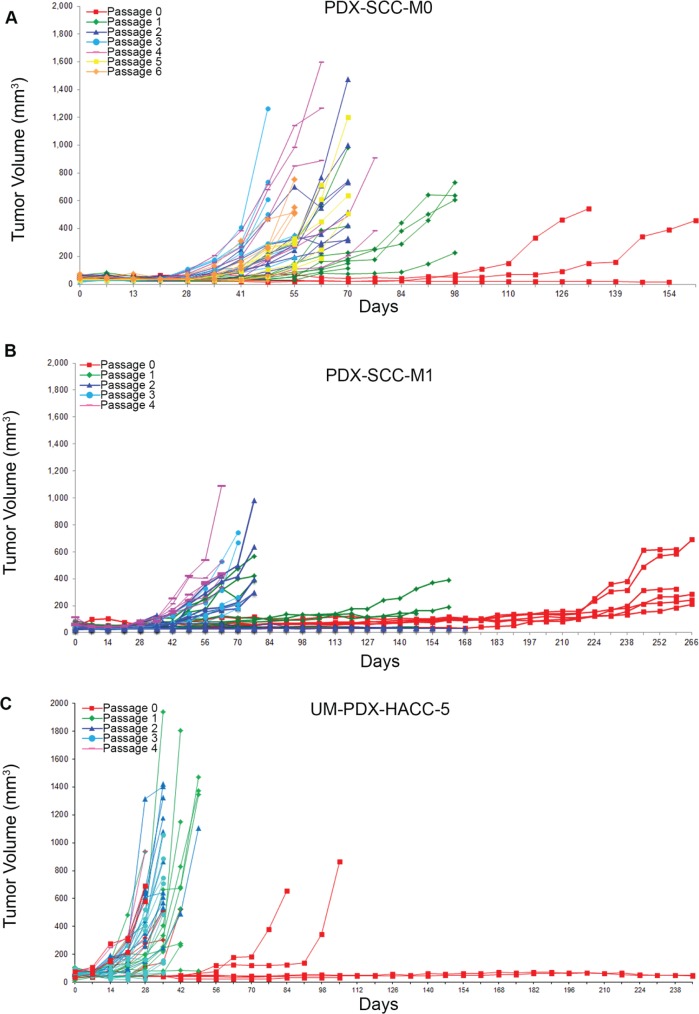
Head and neck cancer PDX models display decreased time to transplantation over passage PDX tumors were grown in SCID mice and tumor size data (mm^3^) is collected over time (days) for sequential passages in three different xenograft models. **A.** PDX-SCC-M0, N = 48 tumors total. **B.** PDX-SCC-M1, N = 30 tumors total. **C.** UM-PDX-HACC-5, N = 55 tumors total. The left-shift between curves of different passages implies a decreasing time between transplantation as passage number increases.

During previous experiments to establish head and neck cancer xenograft models [23], we have consistently observed that the time required to reach harvesting thresholds (1,000-2,000 mm^3^) generally decreases with increased *in vivo* passage. We also observed a general improvement in transplantation yield, with more tumor fragment transplantations resulting in viable tumors in higher passages. We have described tumor growth curves over several *in vivo* passages for PDX-SCC-M0 (Figure 1A), PDX-SCC-M1 (Figure 1B), UM-PDX-HACC-5 (Figure 1C), and UM-SCC-M11 (Supp. Figure 1A). Each of these xenograft models used direct transplantation without sample freezing. The clinical data for each of the models is included in Supplemental Figure 2. For the PDX-SCC-M0 model, the time until harvesting decreased from 266 days at passage 0 to 56 days at passage 6 (Figure 1C). This pattern was observed in both squamous cell carcinoma and adenoid cystic carcinoma, two distinctively different malignancies.

### Tumor growth information across multiple passages can be merged to reflect relative tumor volume over time

**Figure 2:**
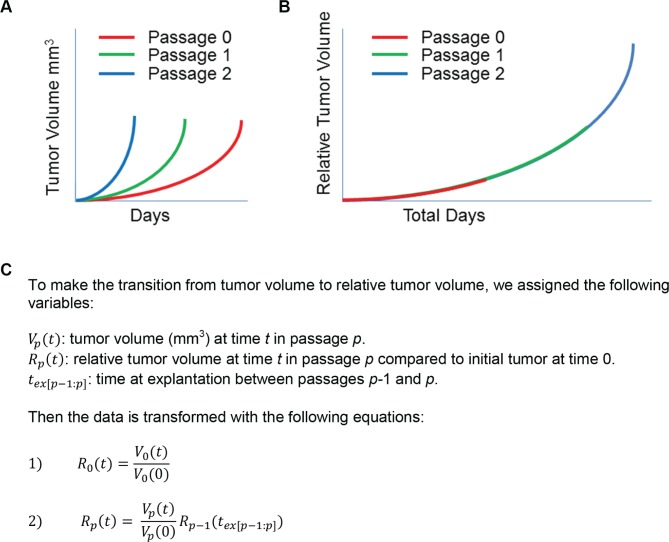
Tumor growth information across multiple passages can be merged to reflect relative tumor volume over time **A.** Representation of tumor volume (mm^3^) over time (days) with multiple passages. **B.** schematic of how this same tumor volume data can be transformed to relative tumor volume, with starting sizes normalized to 1. **C.** Variable assignments and assumptions, with equations used to transform tumor volume to relative tumor volume at each passage.

We aimed to analyze the consistency of growth rates for a single PDX model across multiple passages. In order to accomplish this, we merged data from individual passages (Figure 2A) into a single trajectory across the total age of the PDX model, as opposed to the age of each passage (Figure 2B). Our PDX maintenance protocol calls for implanting fragments of an explanted tumor in order to propagate subsequent xenograft passages. We were concerned that transplanting fragments of different sizes could result in different times to passage, even with similar growth rates. To control this potential bias, we propose that volume measurements are captured as a dimensionless measurement of relative tumor volume size change instead of absolute tumor volume (mm^3^).

We developed a simple set of mathematical equations to transform tumor volume data across different passages into relative tumor volume (Figure 2C). For the original passage, we calculated the relative tumor volume compared to the size of each initial tumor fragment. The relative tumor volume in each passage is then calculated relative to the size of the preceding passage's relative tumor volume at explantation. Careful recording of the tumor pedigree is required to calculate relative tumor volume at a given passage. The transformed data can then be analyzed longitudinally across passages with a single time variable.

To investigate the growth rate of each PDX model over time, we first transformed tumor size data for PDX-SCC-M0, PDX-SCC-M1, PDX-SCC-M11, and UM-PDX-HACC-5 from tumor volume (mm^3^) to relative tumor volume using the equations described in Figure 2C. We assumed exponential growth and therefore log-transformed relative tumor volume. We then used regression analysis to determine if growth rate was constant over time. A repeated-measures mixed effects linear statistical model was fitted to predict log-relative tumor volume. We evaluated the effect of time on relative tumor volume (linear exponential tumor growth rate) and the effect of the interaction of time*time (quadratic exponential tumor growth rate).

In each of our linear models relative tumor volume increased with time, indicating a positive tumor growth rate (Table 1). We included time^2^ to determine if the growth rate was changing over time. In each of the three PDX models we assessed, the growth rate increased over time, as reflected by a positive coefficient to the time^2^ regression term. The time^2^ variable was highly statistically significant. We compared the linear and quadratic models to determine which more accurately fit the data. For all PDX models that we analyzed, the Akaike Information Criterion (AIC) decreased with addition of time^2^.

### PDX models display increasing exponential tumor growth rates

**Figure 3:**
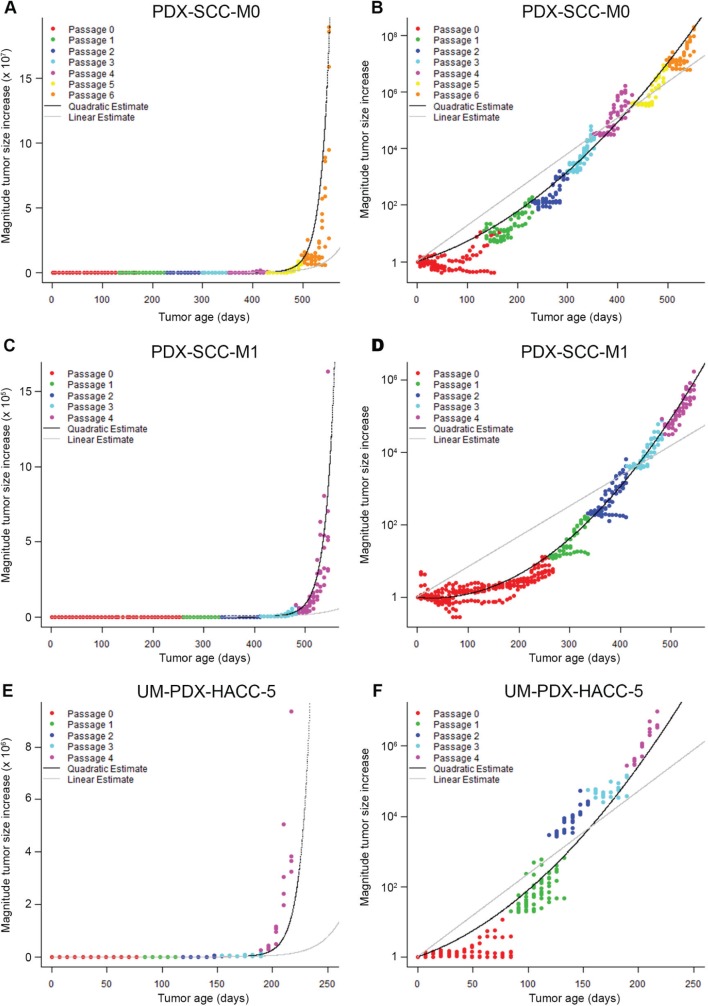
PDX models display increasing exponential tumor growth rates Relative tumor volume versus time is displayed in **A.** and **B.** for PDX-SCC-M0, N = 48 tumors total; **C.** and **D.** for PDX-SCC-M1, N = 30 tumors total; and **D.** and **A.** for UM-PDX-HACC-5, N = 55 tumors total. Prediction lines for linear mixed models including linear time model (grey) and quadratic time model (black) are superimposed. The data is expressed with untransformed axis (left column) and log^­­^_10_-axis (right column). A straight line in the right-hand graphs represents a stable exponential growth rate.

We used the statistical linear mixed models that we fit in Table 1 to generate the linear and quadratic statistical model predictions for each of the PDX models that we analyzed. We plotted the relative tumor volume information versus time for PDX-SCC-M0 (Figure 3A-3B), PDX-SCC-M1 (Figure 3C-3D), UM-PDX-HACC-5 (Figure 3E-3F), and PDX-SCC-M11 (Supp. Figure 1B-1C) lines. We graphically overlayed the predictions for the linear model of time (grey line) and quadratic model of time (black line) on the relative tumor volume data plots. The plots on the left side of Figure 3 represent a linear (untransformed) y-axis, whereas the right side figures have a log-transformed y-axis. Assuming exponential growth, tumors growing at a constant exponential rate should be represented by a straight line on the log-transformed axis. In all cases examined here, the quadratic statistical model fits the data more closely. This agrees with the decrease in AIC we observed (Table 1).

**Table 1:** Statistical regression model output

Xenograft	Time coefficient (*p*-value)	Time^2^ coefficient (*p*-value)	Model AIC
PDX-SCC-M0	0.0291 (< 0.0001)		518.0
	0.0123 (< 0.0001)	0.00004 (< 0.0001)	446.7
PDX-SCC-M1	0.0192 (< 0.0001)		350.4
	−0.0032 (0.0033)	0.00005 (< 0.0001)	263.7
PDX-SCC-M11	0.0150 (< 0.0001)		192.4
	0.0004 (0.8583)	0.00004 (< 0.0001)	181.5
UM-PDX-HACC-5	0.0541 (< 0.0001)		406.9
	0.0248 (< 0.0001)	0.0002 (< 0.0001)	393.4

For PDX-SCC-M0, PDX-SCC-M1, PDX-SCC-M11, and UM-PDX-HACC-5 respectively, we fit linear mixed models for log-relative tumor volume *versus* time. We compared the linear models including time to the quadratic models including time and time^2^. For each of our PDX models, the model fit as measured by AIC improved with the quadratic model.

### Tumor histology changes with increased passage

**Figure 4:**
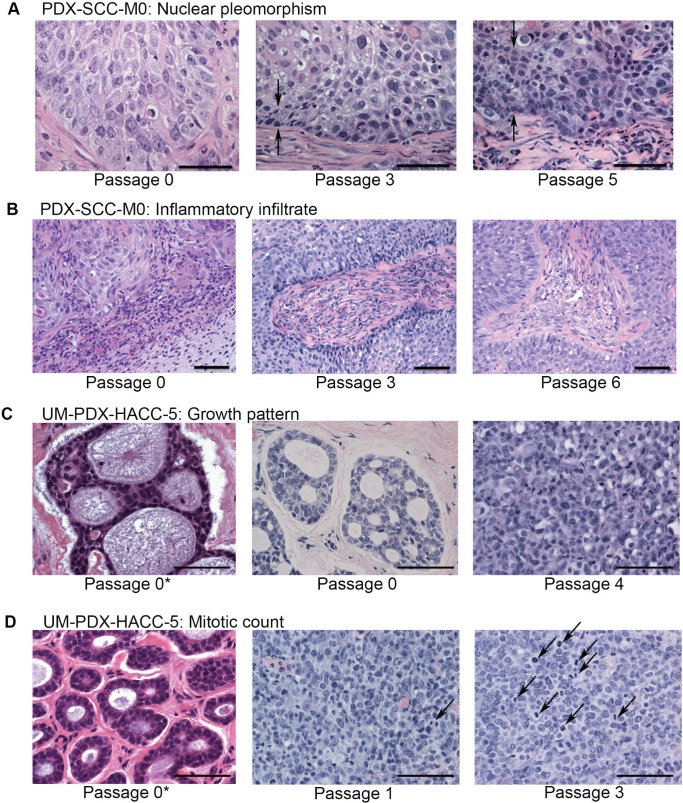
Tumors appearance changes with passage Representative images of hematoxilin and eosin stain of tissue samples of PDX-SCC-M0 in A and B, and UM-PDX-HACC-5 in C and D are shown at different passages. Pictures were taken at x400 magnification in A, C, and D and at x200 magnification in B. Scale bars represent 50 μm in all images. The symbol 0* represents the original patient tumor sample prior to implantation in mouse. **A.** We observed that there appeared to be increasing band of nuclear pleomorphism associated with increased passage number (between arrows). **B.** Decreasing inflammatory infiltrate over passages was also identified. **C.** The adenoid cystic carcinoma growth pattern changed dramatically over passages. **D.** The mitotic count also increased appreciably (arrows mark mitotic figures).

We observed histopathological characteristics at different passages. In squamous cell carcinoma, there was increased nuclear pleomorphism in higher-passage samples compared to lower-passage samples (Figure 4A). We also observed a semi-quantitative decrease in infiltration of inflammatory cells at higher-passage samples compared to lower passage samples (Figure 4B). In adenoid cystic carcinomas, we observed a dramatic change in the overall histopathological pattern associated with increased passage. The tumors at the time of initial excision from the patient presented tubular or cribriform pattern. By the end of the first passage, all tumors had converted to a more-aggressive solid pattern (Figure 4C). We also noted a change in the pattern of mitotic figures based on passage number (Figure 4D).

**Figure 5:**
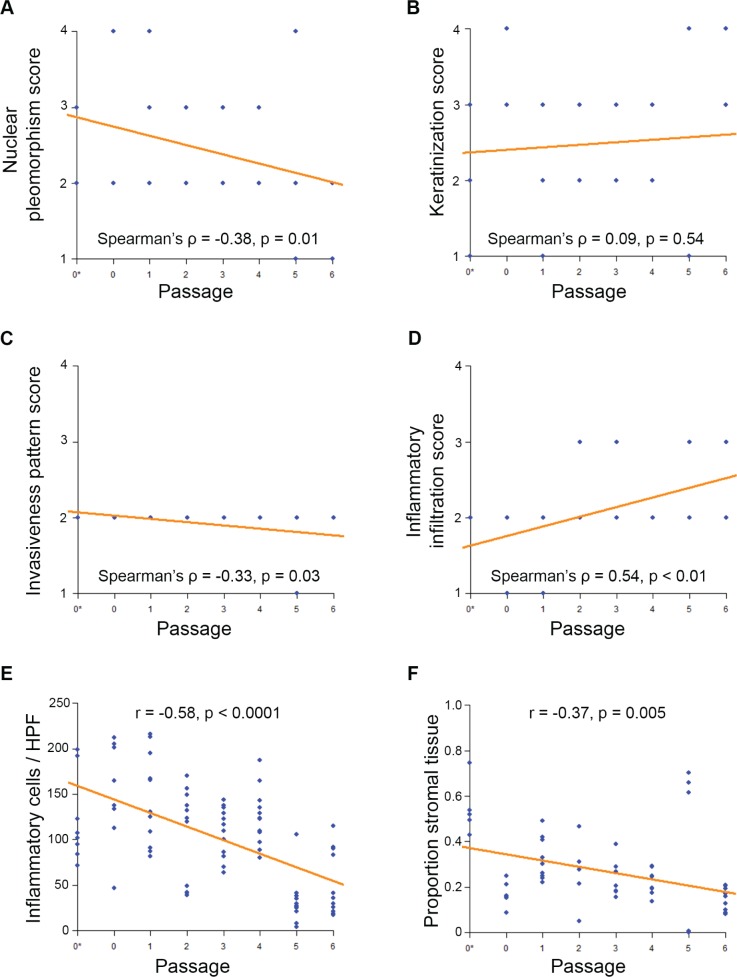
Squamous cell carcinoma PDX models decrease inflammation and stromal tissue with passage Blinded samples from PDX-SCC-M0 were provided to oral pathologists for scoring across passages. N = 57 tissue sample slides. The symbol 0* represents the original patient tumor sample prior to implantation in mouse. **A.** Inflammatory infiltration score **B.** invasiveness pattern score **C.** keratinization score and **D.** nuclear pleomorphism score were compared to passage number. Lower scores represent histopathologically more aggressive characteristics. Correlations were assessed by Spearman's Rank-Correlation. **E.** Inflammatory cell count and **F.** proportion of tumor comprised of stromal tissue were compared to passage number. Correlations were assessed by Pearson Correlation Coefficient.

Objective histopathological rankings changed with increased passage for our squamous cell carcinoma PDX models. We collected hematoxilin and eosin (HE) stained patient and PDX tissue samples for PDX-SCC-M0 cells, and then blinded the samples. Two oral pathologists rated the samples using the Bryne Classification characteristics (Supplemental Figure 3). In this metric, high inflammation is represented by low rank, and high nuclear pleomorphism is represented by low rank. Correlation between rating and passage number was assessed using Spearman's rank correlation coefficient. A significant negative correlation between inflammatory information and increasing passage was observed (Spearman's rho = 0.54, *p* < 0.01) (Figure 5A). A significant positive correlation between nuclear pleomorphism and increasing passage was observed (Spearman's rho = −0.38, *p* = 0.01) (Figure 5D). A positive correlation between passage number and invasiveness was also observed (Spearman's rho = −0.33, *p* = 0.03) (Figure 5B), though there was little variability in this score category. There was no significant correlation between keratinization score and passage number (Figure 5C). Agreement between raters for the Bryne classification was verified using Cohan's Kappa statistic (Cohan's Kappa = 0.727, *p* < 0.0001).

To further explore the passage-related histopathological changes, oral pathologists counted the number of inflammatory cells and stromal tissue proportion within our blinded samples. We used the correlation coefficient to quantify the relationship between variables and passages. There was a strong significant negative correlation between passage number and number of inflammatory cells per blinded high power field (correlation coefficient r = −0.58, *p* < 0.0001) (Figure 5E). There was a significant moderate negative correlation between proportion of stromal tissue and passage number (correlation coefficient r = −0.37, *p* = 0.005) (Figure 5F). No changes were observed in the genotype (Supplemental Figure 4) and single tandem replicate (STR) profiling (data not shown) on PDX-SCC-M0 and PDX-SCC-M1 models at different passages.

**Figure 6:**
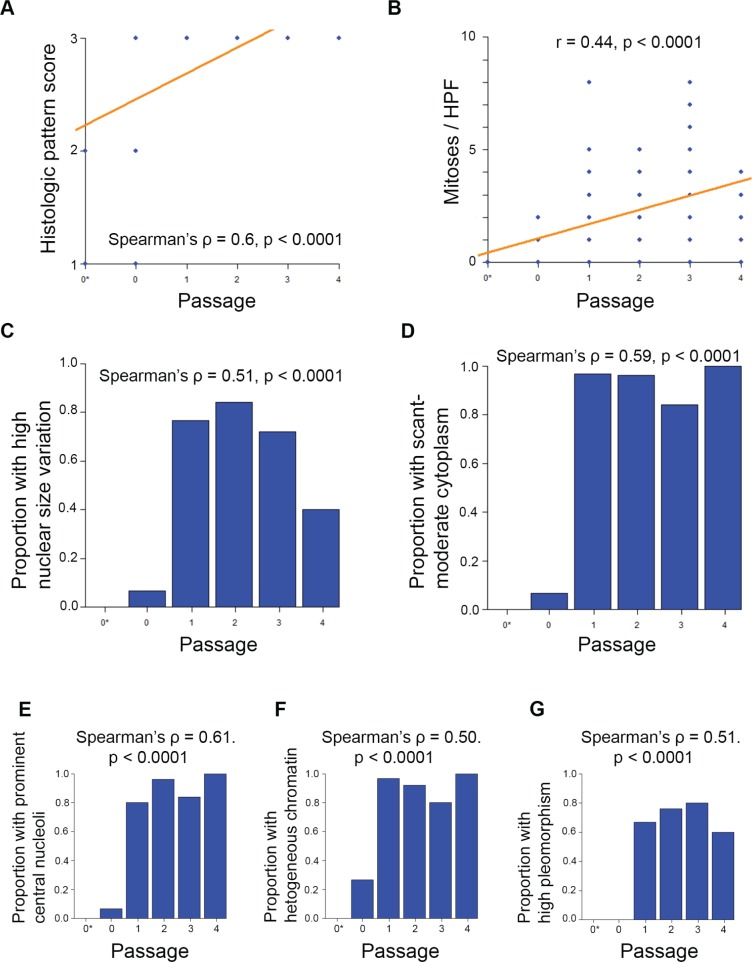
Adenoid cystic carcinoma PDX models change histopathological pattern with passage Blinded samples from UM-PDX-HACC-5 were provided to oral pathologists for scoring across passages. The symbol 0* represents the original patient tumor sample prior to implantation in mouse. N = 111 tissue sample slides. **A.** Histopathological pattern changed from either cribriform or tubular to solid by passage 1. Correlation was assessed by Spearman's Rank-Correlation ρ. **B.** Number of mitoses per x400 magnification high power field by passage. Correlation was assessed by Pearson's correlation coefficient r. Proportion of measured samples in higher-aggressiveness categories were assessed by passage for **C.** high nuclear size variation; **D.** scant or moderate cytoplasm; **E.** prominent central nucleoli; **F.** heterogeneous chromatin; **G.** high pleomorphism. Higher scores represent histopathologically more aggressive characteristics. Correlations were assessed by Spearman's Rank-Correlation ρ.

Histopathological rankings changed with passage number for the adenoid cystic carcinoma PDX model. We evaluated HE-stained patient and PDX samples for UM-PDX-HACC-5, and then blinded the slides. Two oral pathologists rated them using Seethala's ACC rating (Supplemental Figure 5). We identified significant changes to the histopathological pattern of the ACC samples, with complete conversion to the more-aggressive solid appearance by the end of passage 1 (Spearman's ρ = 0.6, *p* < 0.0001) (Figure 6A). We also found that the number of mitoses per high power field was significantly associated with passage number (*r* = 0.44, *p* < 0.0001) (Figure 6B). There was also a significant association between passage number and the proportion of samples with the following components of Seethala's ACC rating: high nuclear size variation, scant-moderate cytoplasm, prominent central nucleoli, heterogeneously dispersed chromatin, and high overall pleomorphism (Spearman's ρ = 0.5-0.61, *p* < 0.0001) (Figure 6C-6G). These findings are compatible with the high-grade transformation in ACC (Supplemental Figure 5). Genotype and STR profiling (data not show) showed no changes across increasing *in vivo* passages.

## DISCUSSION

In this report, we presented a mathematical analysis of PDX tumor growth over time and across sequential xenograft passages. Our results show that the growth rates of PDX models are not constant, and instead accelerate as a function of time. We also showed that in addition to growth rate change, the tumors we investigated showed several histopathological alterations with time. Here, we propose a novel data transformation analysis that translated absolute tumor size to relative tumor volume at time of implantation. We deployed this development to analyze PDX models over multiple passages. In addition, this technique could also be employed to analyze tumor size information across time periods longer than a mouse life, as in development of treatment strategies for indolent cancers. This could also aide in analysis of more subtle differences between antineoplastic treatment classes, such as for treatments that are hypothesized to slow cancer growth for long periods of time.

The data transformation allowed us to determine if rate of tumor size change was constant or dynamic over time. We found that a quadratic equation for the time effect provided a superior data fit to the linear form across four different PDX models. This implies that the rate of exponential tumor growth is increasing as time (*in vivo* passage) increases. We note that quadratic modeling illustrates superiority over linear modeling, but it is unlikely that the growth rate for PDX tumors would continue to increase infinitely as a quadratic function implies. With acquisition of additional tumor growth data, our future work will focus on identification of a superior functional form for describing the long-term behavior of PDX models growth rate change. Our current work is limited to tumor size data grown under identical conditions.

While previous research has supported genomic stability of PDX models at increasing passages, we identified a significant histopathological change across time in both squamous cell carcinoma and adenoid cystic carcinomas. A higher histopathological grade was strongly associated with increasing passage, as seen in the increased nuclear pleomorphism on SCC samples and more aggressive pattern in ACC samples. Furthermore, we quantified a significant decrease in proportion of stromal tissue as well as a decrease in number of immune cells by passage. We postulate that as the human stromal tissue in the PDX models is replaced by murine stromal tissue, the associated immune response is blunted. The secondary decrease of local macrophage *versus* tumor effect then allows for the tumor to grow more rapidly, as seen in the increased nuclear pleomorphism and mitotic figures. Given the stability of genotype and STR profile, these changes could be from epigenetic modification. This finding within the same tumors over time is important because it could theoretically result in different therapeutic responses by the same tumor at different passages. A limitation in the generalizability of these results to general tumor biology is the immune suppressed murine environment of PDX models does not accurately reflect the milieu within human tumors.

We explicitly evaluated growth rate information for two disparate types of head and neck cancer: squamous cell carcinoma and salivary gland adenoid cystic carcinoma. We were able to show that these two cancer types shared an accelerating growth rate over *in vivo* passaging. The broad differences between ACC and SCC tumors in histopathological and clinical attributes suggest that the ability of PDX models to adapt to their murine host environments is a characteristic shared across numerous tumor types. It is important to better understand if more clinically aggressive tumors form faster growing PDX tumors. The most aggressive tumors, while fastest to develop, may not be representative of the majority of tumors of a given type. Our study is limited in the scope of tumors investigated, given that all of our tumors are from head and neck cancers. It is unclear if PDX growth rate increases occur in different tumor histologies. Further mechanistic studies across a variety of tumor types to further understand the observed changes will enhance our understanding and enable more accurate interpretation of translational drug development using PDX tumor models.

## MATERIALS AND METHODS

### Patient-derived xenograft (PDX) tumor models

Three patients with head and neck squamous cell carcinoma and one patient with salivary gland adenoid cystic carcinoma were recruited and consented using our Head and Neck SPORE consent form (Supplemental Figure 2). Our criteria for PDX models were: A) PDX were passaged for at least 3 times sequentially *in vivo* without freezing, and B) Tissue was available for histopathological analysis at each passage. Tumor fragments were transplanted directly into severe combined immunodeficient mice (CB.17.SCID; Charles River, Wilmington, MA, USA) within 24 hours of surgery. Tumor volume was calculated as tumor maximum length x width^2^ / 2 and was measured every 7 days. Each mouse had 2 tumors implanted bilaterally subcutaneously. Tumor end-point was a volume of 2,000 mm^3^. All procedures were reviewed and approved by our Institutional Review Board and University Committee on Use and Care of Animals.

### Histopathological Interpretation

Tissue sample slides from PDX-SCC-M0 and UM-PDX-HACC-5 tumors and respective primary tumors were stained for hematoxilin and eosin (HE) and examined by two oral pathologists (MDM, FN) under blinded conditions for tumor passage. The SCC tumors were evaluated using the Bryne Classification [24] (Supplemental Figure 3). Likewise, the ACC tumors were examined by the same oral pathologists using the high-grade ACC transformation criteria, as described [25-27] (Supplemental Figure 5). Stromal tissue was assessed as a proportion of total tissue sample by calculating tissue sample areas using NIH ImageJ software [28]. Overall stromal tissue proportion was calculated as a weighted average of multiple images based on pixel quantity per image. Correlation between passage number and histopathological values was quantified by Spearman's Rank-Order Correlation [29] for ordinal data, or by Pearson's Product-Moment Correlation Coefficient [30] for continuous variables. P-values were calculated by using the test versions of these correlations [31, 32]. Inter-rater reliability was calculated using Cohen's κ [33]. All calculations were performed using the statistical computing language R [34].

### Tumor size normalization across passages

To compare tumor growth rates from the same xenograft model across multiple passages, we merged data from multiple passages into a single temporal growth database. To accomplish this, we transformed the size of all tumors from volume (mm^3^) into volume increase relative to initial tumor size. We call this new metric relative tumor volume (Figure 2C). We assumed that tumors were passaged on the same day, though some murine implantation surgery occurred up to 23 hours after surgical removal.

### Tumor growth curve regression analysis

Following the transformation from tumor volume to relative tumor volume, tumor growth was analyzed using linear mixed model regression to analyze the repeated measurements on each tumor [35]. Model fixed effects included either time or time and time^2^ (to represent the additional change of growth rate with each additional day). Nested model random effects included each mouse and the side of mouse used for tumor implantation. For all models a continuous autoregressive correlation structure was used, which assumes more correlated variances among temporally proximate observations [36]. A log-transformation of the outcome variable (relative tumor volume) was used because the tumor volumes grow exponentially. Analysis was performed using the “nlme” package in the statistical software program R v3.1.0. [37]. Model predictions were generated directly from the above models with identical assumptions. Comparison between models of varying complexity was performed using Akaike Information Criterion (AIC) [38]. The AIC is a measure of goodness-of-fit with an adjustment for model complexity, which enables comparison between statistical models with different numbers of variables. A smaller AIC represents a better-fitting model.

## SUPPLEMENTARY MATERIAL FIGURE AND TABLES


