# Supplementary material for: Patient-derived xenograft (PDX) tumors increase growth rate with time
Source: Oncotarget. 2016 Jan 14;7(7):7993–8005. doi: 10.18632/oncotarget.6919 (PMC4884970; doi:10.18632/oncotarget.6919)
Supplement: Supplementary file 1 [file oncotarget-07-7993-s001.pdf]

Patient-derived xenograft (PDX) tumors increase growth rate with time

Supplementary Material

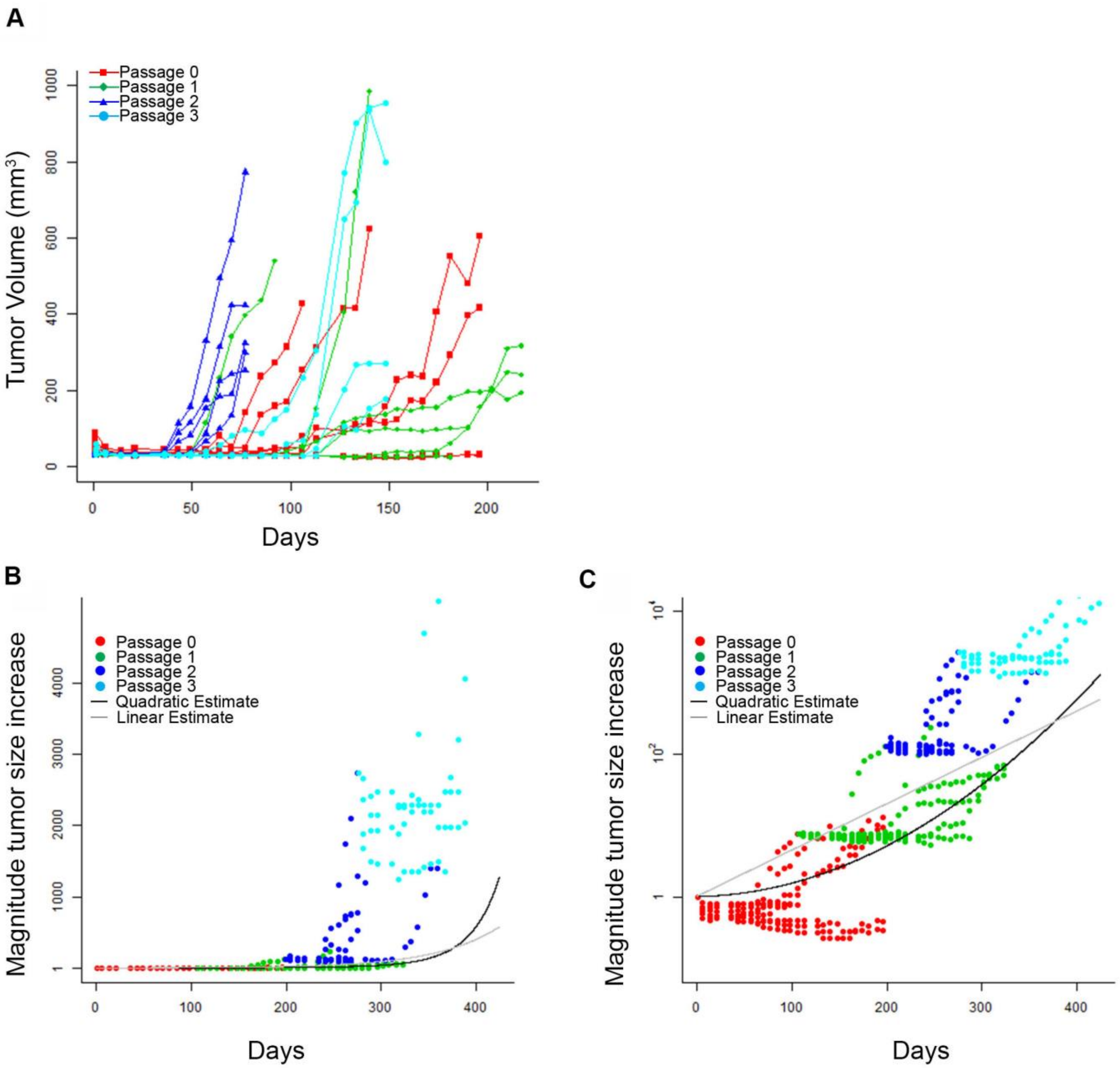

Supplementary Figure 1: A, PDX-SCC-M11 tumors were grown in SCID mice and tumor size data (mm^3) was collected over time (days) for sequential passages (N=24). B and C, Relative tumor volume versus time for PDX-SCC-M11. Prediction lines for linear mixed models including

linear time model (grey) and quadratic time model (black) are superimposed. The data is expressed with untransformed axis (left column) and log_{10}-axis (right column). A straight line in the right-hand graph represents a stable exponential growth rate.

Supplementary Figure 2: Patient characteristics of the PDX models evaluated here

	**PDX-SCC-M0**	**PDX-SCC-M1**	**PDX-SCC-M11**	**UM-PDX-HACC-5**
**Age at diagnosis**	58	72	37	45
**Gender**	Male	Male	Male	Female
**Histologic Subtype**	Squamous cell carcinoma	Squamous cell carcinoma	Squamous cell carcinoma	Salivary gland adenoid cystic carcinoma
**Stage at PDX establishment**	T4aN0M0 (IVa)	T2N1M0 (III)	T4N0M0 (IV)	T4aN0M0 (IVa)
**Perineural Invasion**	Yes	No	Unknown	Yes
**Primary Site**	Unknown	Oral tongue	Sinonasal	Hard palate
**Site of Sample**	Parotid gland	Oral tongue	Sinonasal	Hard palate
**Pre-PDX Radiation**	No	No	Yes	No
**Pre-PDX Chemotherapy**	No	No	Yes	No
**Ablative Surgery**	Parotidectomy and neck dissection	Hemiglossectomy and neck dissection	Skull base dissection	Maxillectomy
**Post-PDX Recurrence**	Yes	No	Yes	No
**Deceased**	No	No	Yes	No

Supplementary Figure 3: Histopathological scoring of head and neck squamous cell carcinomas adapted from the Bryne classification (24)

**Score Value**	**1**	**2**	**3**	**4**
**Keratinization**	Highly keratinized (>50% of cells)	Moderately keratinized (20-50% of cells)	Minimal keratinization (5-20% of cells)	No keratinization (0-5% of cells)
**Nuclear pleomorphism**	Little pleomorphism (>75% of mature cells)	Moderately abundant nuclear pleomorphism (50-75% of mature cells)	Abundant nuclear pleomorphism (25-50% of mature cells)	Extreme nuclear pleomorphism (0-25% of mature cells)
**Pattern of invasion**	Compressive, well delimited infiltrative edges	Infiltrative, solid cords, bands or lines	Small groups or cords of infiltrative cells (n>15)	Remarkable and widespread dissociation in small groups and/or individual cells
**Inflammatory infiltration**	Remarkable	Moderate	Little	Absent

Supplementary Figure 4: Genotypic analysis of DNA was extracted from unfrozen tissues of PDX models studied here

	Chromosome	Gene	Nucleic Acid Change	Amino Acid Change
**PDX-SCC-M0, passage 0**	14	AKT1	G→T	C138A:p.D46E
**PDX-SCC-M0, Passage 5**	14	AKT1	G→T	C138A:p.D46E
**PDX-SCC-M1, passage 0**	4<br>17<br>20	FGFR3<br>TP53<br>GNAS	C→G<br>G→C<br>C→T	C1149G:p.F383L<br>C192G:p.Y64X<br>C556T:p.R186C
**PDX-SCC-M1, passage 2**	4<br>17<br>20	FGFR3<br>TP53<br>GNAS	C→G<br>G→C<br>C→T	C1149G:p.F383L<br>C192G:p.Y64X<br>C556T:p.R186C

Supplementary Figure 5: Histopathological scoring of adenoid cystic carcinomas based on a classification adapted from Seethala and colleagues (26).

**Histopathological Score Value**	**1**	**2**	**3**
**Pattern**	Tubular	Cribriform	Solid

**High Grade Transformation Score Value**	**1**	**2**
**Nucleoli**	Tubular	Cribiform
**Chromatin**	Present but indistinct	Prominent
**Nuclear size**	Uniform size distribution	At least 2-fold nuclear variation
**Cytoplasm**	Scant to nearly absent	Scant to moderate
**Overall pleomorphism**	Low	High
